# Supplementary material for: Dose-dependent action of the RNA binding protein FOX-1 to relay X-chromosome number and determine C. elegans sex
Source: eLife. 2020 Dec 29;9:e62963. doi: 10.7554/eLife.62963 (PMC7787662; doi:10.7554/eLife.62963)
Supplement: Figure 2—source data 1. [file elife-62963-fig2-data1.docx]

**Figure 2—source data 1**

***xol-1* transcription is not repressed by high levels of FOX-1**

|  | | | Transcript measured by qRT-PCR | | | |
| --- | --- | --- | --- | --- | --- | --- |
| Genotype*^a^* | | | *xol-1^b^* | *nhr-64^b^* | *xol-1^c^* | *fasn-1^c^* |
| *yIs44(fox-1); him-5* | vs. | *him-5* | 1.86 ± 0.14 | 1.17 ± 0.10 | 1.80 ± 0.27 | 0.93 ± 0.08 |
| XX & XO |  | XX & XO |  |  |  |  |
|  | | |  |  |  |  |
| *yIs44(fox-1)* | vs. | wild-type | 2.55 ± 0.27 | 1.22 ± 0.11 | 2.20 ± 0.22 | 0.92 ± 0.11 |
| XX |  | XX |  |  |  |  |

*^a^*The levels of *xol-1* transcripts were quantified in embryos of different genotypes to determine if high FOX-1 levels made from *yIs44(fox-1)* altered *xol-1* transcript levels. In the first comparison, *xol-1* transcripts are expressed as the fold change in *yIs44(fox-1); him-5* XX and XO embryos compared to *xol-1* transcripts measured in *him-5* XX and XO embryos. In the second comparison, *xol-1* transcripts are expressed as the fold change in *yIs44(fox-1)* XX embryos compared to *xol-1* transcript levels measured in wild-type XX embryos.

*^b^*The *xol-1* and *nhr-64* transcripts were normalized to the levels of the control gene *fasn-1*, a fatty acid synthase gene whose expression is constant throughout embryogenesis and is not affected by dosage compensation. *nhr-64*, a gene also not affected by dosage compensation, was used as the control to gauge the variability and reliability of measurements made using qRT-PCR. The *fasn-1*-normalized *nhr-64* transcripts were statistically equivalent across the three independent replicates. Experimental error is expressed as the standard error of the mean.

*^c^*Similar results were obtained for the two sets of genotypes in separate qRT-PCR experiments in which transcript levels for *xol-1* and *fasn-1* were normalized to the level of *nhr-64*.

This set of experiments demonstrates that the reduction in *xol-1* activity caused by high levels of FOX-1 is not due to an effect on *xol-1* transcription, since *xol-1* transcript levels in strains with elevated FOX-1 levels are slightly higher, rather than being reduced. This slight elevation in *xol-1* transcript levels in the presence of high FOX-1 might result from the RNA-bound FOX-1 causing increased stability of *xol-1* transcripts, as it does for mammalian transcripts (Lee et al., 2016; Ray et al., 2013). Both the 2.2 kb and 2.5 kb transcripts have GCACG FOX-1 binding sites in their 3' UTRs, and the retained intron VI in the 2.5 kb transcript has both GCAUG and GCACG sites.
