# Supplementary material for: Dose-dependent action of the RNA binding protein FOX-1 to relay X-chromosome number and determine C. elegans sex
Source: eLife. 2020 Dec 29;9:e62963. doi: 10.7554/eLife.62963 (PMC7787662; doi:10.7554/eLife.62963)
Supplement: Figure 6—source data 1. [file elife-62963-fig6-data1.docx]

**Figure 6—source data 1**

**A Overexpression of ASD-1 kills both XO males and XX hermaphrodites**

|  |  | **Total Embryos**  (Broods) | **% XO Viability ± SEM**  Total Adult XO Males | **% XX Viability ± SEM**  Total Adult XX Hermaphrodites |  |
| --- | --- | --- | --- | --- | --- |
|  | |  |  |  | |
| ***him-8; yEx (asd-1+++) isolate 1*** | | **620**  (3) | **32% ± 6**  73 | **19% ± 2**  75 | |
| ***him-8; yEx (asd-1+++) isolate 2*** | | **614**  (3) | **22% ± 3**  51 | **18% ± 5**  72 | |
| ***him-8; yEx (asd-1+++) isolate 3*** | | **709**  (3) | **26% ± 2**  69 | **22%** **± 3**  98 | |

**B FOX-1 family member ASD-1 is unlikely to regulate *xol-1***

|  | **% XX Hermaphrodite**  **Viability ± SEM** | | **XX Hermaphrodite**  **Avg. Brood Size ± SEM** Broods (Total Embryos) |  | **% XX Hermaphrodite**  **Viability ± SEM** | **XX Hermaphrodite**  **Avg. Brood Size ± SEM** Broods (Total Embryos) |
| --- | --- | --- | --- | --- | --- | --- |
|  | | **Empty Vector Control** | |  | ***sex-1(RNAi)*** | |
|  | |  |  |  |  |  |
| **wild type** | | **101% ± 6** | **289 ± 25**  5 (1445) |  | **19% ± 1** | **206 ± 18**  4 (824) |
| ***xol-1(y684)*** | | **99% ± 5** | **301 ± 13**  5 (1505) |  | **84% ± 3** | **207 ± 12**  4 (829) |
| ***fox-1(y793)*** | | **103% ± 6** | **276 ± 15**  5 (1380) |  | **0% ± 0** | **8 ± 2**  8 (66) |
| ***fox-1(y793)***  ***xol-1(y684)*** | | **99% ± 1** | **289 ± 8**  4 (1154) |  | **84% ± 2** | **205 ± 32**  4 (821) |
| ***asd-1(ok2299)*** | | **102% ± 6** | **205 ± 28**  4 (821) |  | **0% ± 0** | **12 ± 3**  8 (98) |
| ***asd-1(ok2299); xol-1(y684)*** | | **98% ± 5** | **266 ± 11**  3 (798) |  | **63% ± 1** | **240 ± 25**  4 (959) |
| ***asd-1(ok2299); fox-1(y684)*** | | **98% ± 2** | **262 ± 17**  5 (1311) |  | **0% ± 0** | **3** **± 1**  8 (21) |

Two independent assays assess whether FOX family member ASD-1 regulates *xol-1*.

(A) Viability of XX and XO *him-8(tm611)* animals carrying extra-chromosomal arrays with multiple copies of wild-type *asd-1* and the dominant roller marker *rol-6*(gf) was assayed to determine whether ASD-1 controls non-productive splicing of *xol-1*. Percent XO viability for each brood from one hermaphrodite was determined by the equation: (total Rol adult males / total embryos) / (0.37). Percent XX viability for each brood from one

hermaphrodite was determined by the formula:  [(total Rol adult hermaphrodites / total embryos) / (0.63)] x 100. The *him-8(tm611)* mutation causes X non-disjunction, resulting in 37% male and 63% hermaphrodite progeny. Average viability from multiple broods and standard error of the mean are shown. Since multiple extra copies of *fox-1* kill XO males but not XX hermaphrodites by causing non-productive *xol-1* splicing, the expectation is that viability of array-bearing XO males should be severely reduced relative to viability of array-bearing XX animals, if ASD-1 promotes non-productive splicing of *xol-1* in XO animals. XO male viability was less reduced compared to XX hermaphrodite viability, providing evidence that ASD-1 does not regulate splicing of *xol-1*.

(B) Viability assays determine whether synergistic lethality occurs from the combination of an *asd-1* mutation and *sex-1(RNAi),* as it does from the combination of a *fox-1* mutation and *sex-1(RNAi).* Viability assays further determine whether synergistic lethality is suppressible by deletion of *xol-1*. As background, XX lethality caused by *sex-1(RNAi)* alone (19% viability) is suppressible to 84% viability in *sex-1(RNAi) xol-1(y684)* mutants (p < 10^-5^). This strong but incomplete suppression indicates that *sex-1* not only represses *xol-1*, but also controls other genes either downstream of *xol-1* in the sex determination / dosage compensation pathway and/or in unrelated pathways.

Further, complete synergistic lethality occurs for *fox-1(y793) sex-1(RNAi)* mutants, but it is also suppressible to 84% viability by a *xol-1* deletion in the *fox-1(y793) xol-1(y684) sex-1(RNAi)* strain (p < 10^-5^). This strong but incomplete suppression reflects two reasons for XX lethality: (1) Reduced function of two X-signal elements kills XX animals by causing elevated *xol-1* expression; (2) *sex-1(RNAi)* kills XX animals due to *xol-1* derepressing and to deregulation of genes other than *xol-1*.

Regarding potential *xol-1* regulation by *asd-1*, our analysis reveals that an *asd-1* mutation causes a different outcome from a *fox-1* mutation. While complete synergistic lethality results from the combination of an *asd-1* mutation and *sex-1(RNAi)*, the lethality is only suppressible to 63% viability by deletion of *xol-1* (p = 1.6 x 10^-4^) for *asd-1* triple mutant vs. *fox-1* triple mutant). If *asd-1* controlled *xol-1*, as does *fox-1*, the XX viability should be restored to 84%. The extra XX lethality results from synergistic effects between the *asd-1* mutation and *sex-1(RNAi)* on targets other than *xol-1*.

Experiments, viability measurements, and brood counts were conducted as described for Figure 6 and Figure 6--figure supplement 1.­­
