# Supplementary material for: Dose-dependent action of the RNA binding protein FOX-1 to relay X-chromosome number and determine C. elegans sex
Source: eLife. 2020 Dec 29;9:e62963. doi: 10.7554/eLife.62963 (PMC7787662; doi:10.7554/eLife.62963)
Supplement: Supplementary file 1. — This table includes a complete list of primers. [file elife-62963-supp1.docx]

**Supplementary File 1. List of primers.**

| **Target** | **Figure or Source Data** | **Sequence** |  | **Function** |
| --- | --- | --- | --- | --- |
| *asd-1* | Figure 6--source data 1 | GATTGAAGGGAGACGTGTTGAAGTGAATCTTGCTACTCAGAGAGTTCAC | BF-2686 | PCR |
|  |  | GGGATATCAGAGCATTTTGAGCACTCATTGCGTCCACTCCAACTG | BF-2687 | PCR, sequencing |
|  |  | CCATGATAGTCTAATTTATGAAATTGCATGCAATGTTTCTCGC | BF-2688 | PCR |
|  |  |  |  |  |
| *dpy-10* | 6 | CGAACGTTCTCGCTGACAACGAACTATTCGCGTCAG | BF-1853 | PCR, sequencing |
|  |  | GCATGTTTGATTTGGAGTAGTTCCTGGCATTCC | BF-1854 | PCR |
|  |  |  |  |  |
| *fox-1* | 6 | GTCAGAAGGAAGAAAACGGAGAAGAAACAGCAGCAACAGCAGAAGAAGC | BF-2492 | PCR, sequencing |
|  |  | CCTCGGCGTTTGGCGAACAATACCTTAGCAACGC | BF-2493 | PCR |
|  |  | GGAAGAATAGGAGTTATCGCGTTGCTGTGAAATGTGATACAATCG | BF-2394 | PCR |
|  |  |  |  |  |
| *xol-1* | 6 | GCACCCAGAAGATTTCACACCACAAATG | BF-2518 | PCR, sequencing |
| *(all but y810)* |  | TGTGACACATGGCGTTAATTACAATAGATACTG | BF-2519 | PCR |
|  |  |  |  |  |
| *xol-1(y810)* | 6 | CACTCTTCATCCTCATCATACGTGTCATCTTGTCGAGCACTTGGAGC | BF-2301 | PCR, sequencing |
|  |  | CGATATTCTAGTTCCAGCATATATGACGGCTCATTCCAAGAACCGTG | BF-2676 | PCR |
|  |  | CAAAATGCATATTTGATCGAATGCCTGCACGTTTGACG | BF-2746 | PCR |
|  |  |  |  |  |
| *xol-1 repair templates* |  | GACTCTAGTGGCAAACTTGCCGTCATC  ACAGACTGTTACAATGACACAACTCTC | BF-2507  BF-2508 | PCR  PCR |
|  |  |  |  |  |
| *asd-1* | Figure 6--source data 1 | AGATTTGATCATTTTGTGCAGGAACTCCTTCGTTATTTGCCTGGACTAC | BF-2748 | PCR |
|  |  | GATGTGCAGCTATTTTGAGATTTCCGATGCCTGATTTAGATGATGAGCCGATGGATG | BF-2749 | PCR |
|  |  |  |  |  |
| *xol-1*  intron VI | 5 | CAAAAUUGCAUGUAGCACAUUUGAUCGUUAUGCUUGCACGCAAAC | B-45 | RNA oligo for binding/competition |
|  |  | GCAUGUAGCACAUUUGAUCGUUAUGCUUGCACGCAAA | B-37 | RNA oligo for binding/competition |
|  |  | CAUUUGAUCGUUAUG | B-15 | RNA oligo for binding/competition |
|  |  | GCAUGAGUUCAUUUGAUCGUUAUGAUGCACGGAAA | C-35 | RNA oligo for binding/competition |
|  |  | CAAAAUGCAUAUUUGAUCGAAUGCCUGCACGUUUG | E-35 | RNA oligo for binding/competition |
|  |  | GCAUAUUUGAUCGAAUGCCUGCACG | E-25 | RNA oligo for binding/competition |
|  |  |  |  |  |
| *xol-1* |  | GCAGGTTGAAGCAAATTCTGAGAGAAG | *xol-1 -* | PCR of *xol-1* isoforms |
|  |  | CACTCTTCATCCTCATCATACGTGTC | *xol-1 +* | PCR of *xol-1* isoforms |
|  |  |  |  |  |
| *xol-1* |  | GAGTTTGATAGCCAAGTTGCTCTTG | CSNP-8 | RT-PCR |
|  |  | AAGCAGTGGTATCAACGCAGAGTAC(T)30(N-1)(N), where (N-1) is A,C, or G and (N) is A, C, G, or T | 3' RACE primer | RT-PCR |
|  |  | CATGAGCAAGTAGAAGGTTTCGAAG | CSNP-9 | RT-PCR |
|  |  | AAGCAGTGGTATCAACGCAGAGT | 3' common | RT-PCR |
|  |  | AATCAGGCCACGGCGCTAATCACG | CSNP-12 | RT-PCR |
|  |  | CGCTGGATCAAATCTGTCGATCC | CSNP-13 | RT-PCR |
|  |  |  |  |  |
| *fasn-1* | Figure 2—data source 1 | GATCCATTTGCAACTGATTCC | *fasn-1 +* | qPCR |
|  |  | GCTTGGTAAGGATGGTGGC | *fasn-1 -* | qPCR |
|  |  |  |  |  |
| *nhr-64* | Figure 2—data source 1 | TAGAGGAAATGCGACAACGG | *nhr-64 +* | qPCR |
|  |  | CCCTCATTTGGTAGCATCAG | *nhr-64 -* | qPCR |
