# Supplementary material for: Dose-dependent action of the RNA binding protein FOX-1 to relay X-chromosome number and determine C. elegans sex
Source: eLife. 2020 Dec 29;9:e62963. doi: 10.7554/eLife.62963 (PMC7787662; doi:10.7554/eLife.62963)
Supplement: Supplementary file 2. — This table lists the gene targets, the target-specific sequence for each Cas9 guide RNA, the figure in which the results are presented, the genomic coordinates corresponding to each guide RNA, and the reference name of each guide. Two guides were used together to delete the fox-1 gene, and two guides were used together to replace sequences within intron VI of xol-1. Coordinates are based on Wbcel235/c11 version of the C. elegans genome. [file elife-62963-supp2.docx]

**Supplementary File 2. List of target-specific sequences for guide RNAs used in CRISPR / Cas9 genome editing experiments**

| **Target** | **Figure** | **Target-specific guide sequence (5’ to 3’)** | **Coordinates** | **Guide name** |
| --- | --- | --- | --- | --- |
| *dpy-10* | 6 | GCUACCAUAGGCACCACGAG | II: 6711193..6711212 | crispr_bf32 |
|  |  |  |  |  |
| *fox-1* | 6 | GAAGUGGUGGCGAGCGUGGA | X: 2456032..2456051 | crispr_bf66 |
| *fox-1* | 6 | UCGAUUUACUCCGUAUUGAG | X: 2446074..2446093 | crispr_bf67 |
|  |  |  |  |  |
| *xol-1* | 6 | GAAUGAGCCGUCAUAUAUGC | X: 8042727..8042746 | crispr_bf68 |
| *xol-1* | 6 | UAGCUAUUGCUACUGAAUCA | X: 8043398..8043417 | crispr_bf69 |
|  |  |  |  |  |
