# Supplementary material for: Dose-dependent action of the RNA binding protein FOX-1 to relay X-chromosome number and determine C. elegans sex
Source: eLife. 2020 Dec 29;9:e62963. doi: 10.7554/eLife.62963 (PMC7787662; doi:10.7554/eLife.62963)
Supplement: Supplementary file 3. — This table presents information for CRISPR/Cas9 genome editing experiments involving HDR using single-stranded repair templates. The table lists the gene targets that were edited, the figure in which the results are presented, the DNA sequences of the repair template, with SNPs highlighted in red letters and deletion junctions highlighted in blue letters, the reference names of related guides, and the reference names of the oligonucleotide repair template. Two guide RNAs were used together in combination with the repair template to delete the endogenous fox-1 gene, resulting in the allele fox-1(y793). The double-stranded repair templates used for inducing mutations in the DNA encoding intron VI of endogenous xol-1 are available upon request but are too long to include in this table. [file elife-62963-supp3.docx]

**Supplementary File 3. DNA sequences of repair templates used for CRISPR / Cas9 genome**

**editing experiments**

| **Target** | **Figure** | **Sequence of repair template (5’ to 3’)** | **Related guide** | **Oligo name** |
| --- | --- | --- | --- | --- |
| *dpy-10* | 6 | CACTTGAACTTCAATACGGCAAGATGAGAATGACTGGAAACCGTACCGCatGcGGTGCCTATGGTAGCGGAGCTTCACATGGCTTCAGACCAACAGCCTAT | crispr_bf32 | BF-1813 |
| *fox-1* | 6 | CCGTTTCCGACACGACGACTGACTCTTCAGTCAGTCATTCCTAGCCGTCCGAGTGGCCCCCCGTTTTGGTAAACGATATTCAATTCCTGACCTCACTTTT | crispr_bf66 | BF-2480 |
|  |  |  | crispr_bf67 |  |
|  |  |  |  |  |
