# Supplementary material for: Dose-dependent action of the RNA binding protein FOX-1 to relay X-chromosome number and determine C. elegans sex
Source: eLife. 2020 Dec 29;9:e62963. doi: 10.7554/eLife.62963 (PMC7787662; doi:10.7554/eLife.62963)
Supplement: Supplementary file 4. — The table lists the gene targets, the figure or table in which the results are presented, the sequences of oligonucleotides used to screen for CRISPR/Cas9 induced mutations or to determine sequences of resulting mutations, the reference name of each oligonucleotide, and the function of each oligonucleotide. For asd-1, the oligos were used to verify the construction of strains built with a pre-existing asd-1 mutation, not for identifying new mutations made using Cas9. The oligonucleotides BF-2507 and BF-2508 were used to synthesize xol-1 repair templates rather than to identify Cas9-induced mutations in intron VI. [file elife-62963-supp4.docx]

**Supplementary File 4. List of oligonucleotides used to screen for CRISPR / Cas9 induced mutations and to determine sequences of resulting mutations**

| **Target** | **Figure or Source Data** | **Sequence** | **Oligo name** | **Function** |
| --- | --- | --- | --- | --- |
| *asd-1* | Figure 6--source data 1 | GATTGAAGGGAGACGTGTTGAAGTGAATCTTGCTACTCAGAGAGTTCAC | BF-2686 | PCR |
|  |  | GGGATATCAGAGCATTTTGAGCACTCATTGCGTCCACTCCAACTG | BF-2687 | PCR, sequencing |
|  |  | CCATGATAGTCTAATTTATGAAATTGCATGCAATGTTTCTCGC | BF-2688 | PCR |
|  |  |  |  |  |
| *dpy-10* | 6 | CGAACGTTCTCGCTGACAACGAACTATTCGCGTCAG | BF-1853 | PCR, sequencing |
|  |  | GCATGTTTGATTTGGAGTAGTTCCTGGCATTCC | BF-1854 | PCR |
|  |  |  |  |  |
| *fox-1* | 6 | GTCAGAAGGAAGAAAACGGAGAAGAAACAGCAGCAACAGCAGAAGAAGC | BF-2492 | PCR, sequencing |
|  |  | CCTCGGCGTTTGGCGAACAATACCTTAGCAACGC | BF-2493 | PCR |
|  |  | GGAAGAATAGGAGTTATCGCGTTGCTGTGAAATGTGATACAATCG | BF-2394 | PCR |
|  |  |  |  |  |
| *xol-1* | 6 | GCACCCAGAAGATTTCACACCACAAATG | BF-2518 | PCR, sequencing |
| *(all but y810)* |  | TGTGACACATGGCGTTAATTACAATAGATACTG | BF-2519 | PCR |
|  |  |  |  |  |
| *xol-1(y810)* | 6 | CACTCTTCATCCTCATCATACGTGTCATCTTGTCGAGCACTTGGAGC | BF-2301 | PCR, sequencing |
|  |  | CGATATTCTAGTTCCAGCATATATGACGGCTCATTCCAAGAACCGTG | BF-2676 | PCR |
|  |  | CAAAATGCATATTTGATCGAATGCCTGCACGTTTGACG | BF-2746 | PCR |
| *xol-1 repair templates* |  | GACTCTAGTGGCAAACTTGCCGTCATC  ACAGACTGTTACAATGACACAACTCTC | BF-2507  BF-2508 | PCR  PCR |
